# Polθ Inhibition: An Anticancer Therapy for HR-Deficient Tumours

**DOI:** 10.3390/ijms24010319

**Published:** 2022-12-24

**Authors:** Gabriela Barszczewska-Pietraszek, Małgorzata Drzewiecka, Piotr Czarny, Tomasz Skorski, Tomasz Śliwiński

**Affiliations:** 1 Laboratory of Medical Genetics, Faculty of Biology and Environmental Protection, University of Lodz, 90-236 Lodz, Poland; 2 Department of Medical Biochemistry, Medical University of Lodz, 92-216 Lodz, Poland; 3 Fels Cancer Institute for Personalized Medicine, Lewis Katz School of Medicine, Temple University, Philadelphia, PA 19140, USA

**Keywords:** Polθ inhibitors, anticancer treatment, DNA double-strand break repair, DNA repair enzyme

## Abstract

DNA polymerase theta (Polθ)-mediated end joining (TMEJ) is, along with homologous recombination (HR) and non-homologous end-joining (NHEJ), one of the most important mechanisms repairing potentially lethal DNA double-strand breaks (DSBs). Polθ is becoming a new target in cancer research because it demonstrates numerous synthetically lethal interactions with other DNA repair mechanisms, e.g., those involving PARP1, BRCA1/2, DNA-PK, ATR. Inhibition of Polθ could be achieved with different methods, such as RNA interference (RNAi), CRISPR/Cas9 technology, or using small molecule inhibitors. In the context of this topic, RNAi and CRISPR/Cas9 are still more often applied in the research itself rather than clinical usage, different than small molecule inhibitors. Several Polθ inhibitors have been already generated, and two of them, novobiocin (NVB) and ART812 derivative, are being tested in clinical trials against HR-deficient tumors. In this review, we describe the significance of Polθ and the Polθ-mediated TMEJ pathway. In addition, we summarize the current state of knowledge about Polθ inhibitors and emphasize the promising role of Polθ as a therapeutic target.

## 1. Introduction

One of the hallmarks of cancer cells is their genetic instability, which could lead to an increase of mutations in their genomes [1]. As a consequence, the loss of function mutations may take place in the genes that are crucial for cell survival mechanisms, for example, DNA repair systems. Under such conditions, the survival of cancer cells depends on finding a substitute for the lost pathway [2]. If inactivation of a specific set of genes leads to cell death, whereas inactivation of each of these genes individually does not affect cell functioning and survival, then these genes are considered to exhibit “synthetic lethal” interactions [3]. Targeting alternative pathways using inhibitors against DNA double-strand breaks (DSBs) repair proteins is becoming a feasible strategy that has been gaining increasing interest in recent years. An approach based on synthetic lethality might not only prove to be a selective and effective solution in personalized anticancer therapy, but it is already contributing to expanding the knowledge about genetic interactions occurring in cells [4,5]. 

DNA polymerase theta (Polθ) is encoded by *POLQ*—a unique multifunctional replication and repair gene that encodes a protein with N-terminal superfamily 2 helicase domain exhibiting ATPase activity and C-terminal A-family polymerase domain [6,7]. The possession of helicase domain is a unique Polθ feature among other eukaryotic DNA polymerases. More detailed information about structure and function of Polθ can be found in another paper of Drzewiecka et al. (2022) [4]. Polθ overexpression has been identified in a number of human cancers and has been linked with a poor clinical outcome for liver cancer and breast cancer patients with homologous recombination (HR) deficiency [8,9,10]. To target DNA repair vulnerabilities in cancer, Zatrenau et al. (2021) [11] discovered nanomolar potent, selective, low molecular weight, allosteric inhibitors of Polθ ART558 and ART812, which interact with the polymerase domain. ART558 inhibits the major Polθ-mediated DNA repair process, i.e., Polθ-mediated end joining (TMEJ) without targeting non-homologous end joining (NHEJ) [11]. Recently, another biochemical compound, RP-6685, with potential to inhibit Polθ polymerase domain was discovered [12]. Additionally, an antibiotic, novobiocin (NVB), was identified as the inhibitor of Polθ helicase activity [13].

The application of Polθ Inhibitors (Polθi) in the concept of dual synthetic lethality emerged after initial success of PARP inhibitors (PARPi) when it was found that tumor cells do not respond to one drug treatment and develop resistance [14]. Polθ has a particular importance for the repair of DSBs in cancer cells deficient in the HR function. Polθ inhibition boosts the effect of PARPi by exerting a synthetically lethal action on BRCA1- and BRCA2-mutant cancer cells [3,14]. Deficiencies in genes of other DNA damage response (DDR) pathways, e.g., encoding DNA-PKcs which is a crucial component of the classical NHEJ pathway, can also make Polθ a key factor for cellular survival [15]. Furthermore, knocking out *POLQ* in mouse models and non-cancerous cells had minimal effect [16,17]. Therefore, Polθ shows promising results as an antitumor drug target candidate, principally against HR-deficient tumors. Moreover, Polθ inhibitors not only have clinical potential in targeting BRCA-gene defective cancers but could also be used to target PARPi resistance [11,13,14]. 

A review of literature focused on the role of polymerase theta in the context of synthetic lethality and potential anticancer therapy was conducted, using PubMed and Google Scholar to search. The authors considered studies performed on animals as well as human subjects (in vivo and in vitro) along with clinical trials. Keywords applied were as follows: DNA polymerase theta, polymerase theta inhibitors, ART558, novobiocin, microhomology-mediated end joining, MMEJ, DNA repair, cancer, polymerase theta-mediated end joining, TMEJ, double strand break repair, homologous recombination repair, HR, non-homologous end joining, NHEJ, siRNA, shRNA, RNA interference, CRISPR/Cas9, anticancer therapy, and synthetic lethality.

## 2. The Role of Polθ—Mediated TMEJ

Polθ is a main protein of TMEJ which is one of the main pathways of DSB repair [7,18]. TMEJ could be considered a substitute pathway to NHEJ and placed side by side with microhomology-mediated end joining (MMEJ) or alternative end-joining (a-EJ) as they share a requirement for microhomology fragments [14,19,20]. However, some publications differentiate TMEJ as a separate pathway, alongside NHEJ, HR, and SSA, considering the existence of a-EJ pathway without Polθ activity. Therefore, in this review the the term “TMEJ” for Polθ-mediated repair process is used, even though it is often called “a-EJ”, “alt-NHEJ”, etc. in the literature [4,21,22,23,24]. 

**Figure 1:**
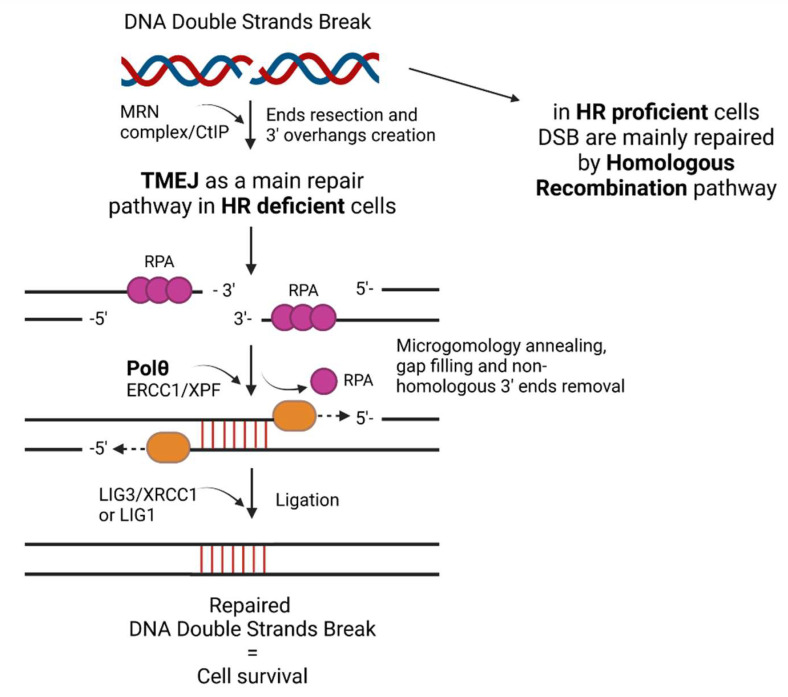
The mechanism of DNA double strand break repair by TMEJ in HR-deficient cells.

TMEJ is determined by several factors, namely: independence on Ku, XRCC4 and LIG4 proteins, resected DNA ends with 3′ single-stranded overhangs, several nucleotide-long microhomology regions and presence of Polθ [21]. Moreover, this repair is highly error-prone due to the lack of Polθ proofreading ability and deleterious characteristics of microhomology end-joining itself. This results in the accumulation of mutations [6]. On the other hand, in HR-deficient tumor cells, where Polθ is usually overexpressed, TMEJ enables their survival (Figure 1) [8,9,10,22,25,26,27]. In some studies, it was observed that TMEJ is most crucial when HR and NHEJ are not working properly [24,25]. However, there is evidence that it is active also in NHEJ-proficient cells [28].

Further, going into details of repair mechanism in the first step of the process, the CtIP with MRN complex is needed to initiate end resection and create 3′ overhangs. It is assumed that PARP1 is involved in the recognition of DNA breaks and helps in end resection [19,25,29]. Subsequently, non-homologous 3′ ends are removed by ERCC1/XPF nucleases. Then, Polθ attaches to single-stranded DNA (ssDNA) overhangs and anneals the sequences based on at least 2 bp microhomology [18,29,30]. In this step the helicase domain of Polθ removes RPA from ssDNA tails, while the polymerase domain is responsible for annealing [7,14]. Therefore, both helicase and polymerase domains of Polθ are necessary in cis configuration for TMEJ to function [21]. Eventually, LIG3-XRCC1 complex or LIG1 ligate stabilized DNA ends [18,25,30]. 

Furthermore, it is believed that Polθ can participate in other repair mechanisms and cell events, such as base excision repair, mismatch repair, replication-associated DNA breaks, or reverse transcription and translation synthesis [6,20,31,32]. However, this is not the subject of this review and further information can be found in the work of Drzewiecka et al. (2022) [4].

## 3. Different Strategies for Polθ Suppression

The consequences of Polθ inhibition and knockdown in cells have been vastly described in the literature [7,9,28,31,33,34,35,36], allowing to evaluate the significance of the protein and its interactions [8]. In the literature, the most used methods include siRNA or shRNA silencing and CRISPR-Cas9 technique, shown by research examples described below. Two other gene editing tools are also described, namely ZFNs (zinc finger nucleases) and TALENs (Transcription activator-like effector nucleases). However, they are considered less efficient and are less frequently used than RNA interference (RNAi) and CRISPR/Cas9, at least in the context of Polθ research [37,38,39]. In this chapter, the authors will review recent research papers that describe the above-mentioned methods of Polθ inhibition and their consequences.

### 3.1. RNA Interference Technique—siRNA and shRNA

#### 3.1.1. Description of the Technique

The main objective of RNA interference is to selectively silence a gene via non-coding RNA which targets and triggers degradation of mRNA. Almost 20 years have passed since the first such molecule was discovered, i.e., microRNA (miRNA), further resulting with the Nobel prize for Fire and Mello in 2006 for defining RNA interference and its mechanism [40,41,42]. Based on this achievement, scientists designed other RNA molecules, and two the most common are siRNA and shRNA [41,43].

Gene silencing can be achieved in two ways: by degradation of the target mRNA induced by small interfering RNAs (siRNAs) or short hairpin RNAs (shRNAs) and differently via suppression of specific mRNAs translation induced by miRNA. This paper will focus on the first approach, achieved with siRNA or shRNA. The molecules lead to a similar genetic outcome, however they are different in terms of structure and molecular mechanism, and may have distinct applications [44].

**Figure 2:**
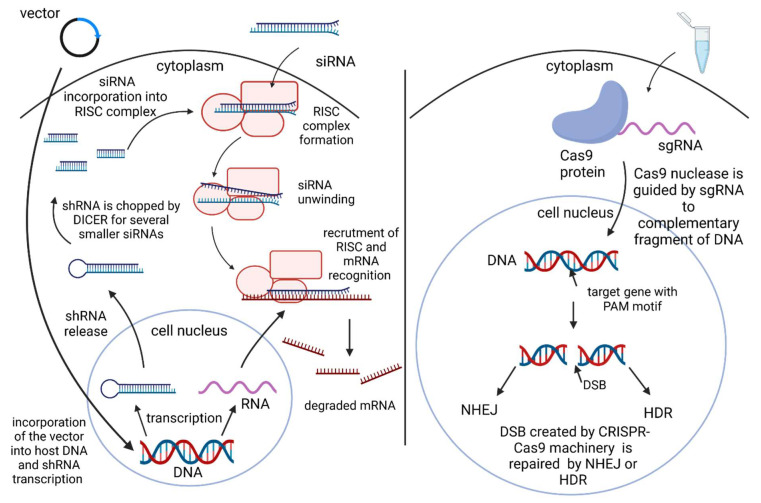
The RNA interference mechanism and CRISPR-Cas9 mechanism in human cell.

siRNAs are double-stranded RNA molecules which total length is 21–25 nucleotides. Along with piwi-interacting RNAs (piRNAs) and miRNAs, siRNAs are defined as non-coding, small RNAs [45]. Considering the structure, siRNAs have one guiding strand (antisense) and a passenger strand (sense), as well as two 2-nucleotide-long overhangs at 3′ ends. [41,46]. siRNA is formed from long double-stranded RNA, cleaved by Dicer, an enzyme from RNase III family. For the purpose of therapy, siRNAs are synthesized chemically and delivered in various ways to the cytoplasm, i.a.: nanocarriers, aptamers, and antibodies [41,44,47]. In the cytoplasm, siRNA creates the RNA-induced silencing complex (RISC) with proteins Dicer, Argonaute-2 (Ago2), and Trans-activating Response RNA Binding-Protein (TRBP), which later allows siRNA to target mRNA. In this interaction, Ago2 splits the sense strand of the molecule, unwinds the duplex with the use of the Dicer N-helicase domain and leads to the degradation of this strand. Then, the anti-sense strand guides the activated RISC complex to target mRNA with its complementary sequence (Figure 2) [41,46]. 

Opposite to the siRNA, shRNA needs to be introduced to the cell’s nucleus. This could be achieved through a bacterial or viral vector. However, a viral vector is most commonly used, as it is considered more stable and efficient [48]. Usage of a viral vector allows shRNA to be integrated to the genome of host cells and later expressed in the nucleus. Afterwards, the host’s protein exportin 5 is responsible for transferring the shRNA outside the nucleus. In the cytoplasm, it forms a complex with Dicer, an RNase III enzyme, which chops up the shRNA into small siRNA duplexes with 20–25 nt of length and 2 nt overhangs at the 3′ end. Then, the siRNAs follow the regular path to degrade desired mRNA (Figure 2) [42,48]. 

RNAi is a promising technology for the treatment of various diseases such as cancer, viral infections, eye and liver diseases, and some genetic disorders. In many applications, siRNA-based therapies are at the stage of clinical trials [41]. In addition, various studies show that both siRNA and shRNA are effective in vivo with different targets and exhibit potential in personalized therapies [40,44,46]. Moreover, according to Alshaer et al. (2021) [41], siRNA could be a better therapeutic tool than small molecules, since it is highly selective, can reach the target in any location and has only antagonistic effects. 

Although it theoretically seems to be a perfect method to perform gene knockdown, there are several drawbacks in practice which should be addressed, such as the way in which RNAi is delivered, off-targets and stabilization of molecules inside cells. Considering these factors, shRNA is regarded to be more efficient than siRNA. When comparing these two molecules, shRNA tends to be more effective inside cells, because it can be synthesized constantly [44]. However, the use of shRNA with a vector could be more complicated and time-consuming [48]. Researchers are still working on improving this method, for example, by means of designing bi-functional shRNA that combines two types of shRNAs are cleaved by RISC-dependent and -independent pathways, thereby leading to gene silencing by mRNA degradation and translation inhibition at the same time [44].

#### 3.1.2. Application in Studies 

Several studies using siRNA and shRNA to silence *POLQ* show successful Polθ mRNA depletion [8,9,35,36]. The studies of Dai et al. (2016) [35] and Kelso et al. (2019) [36] on cancer cell lines confirm increased sensitivity of cells to cisplatin after *POLQ* silencing with siRNA.

Moreover, in a research study that involved inhibiting Polθ via siRNA, Ceccaldi et al. (2015) [8] presented its correlation with HR repair mechanism. The authors concluded that Polθ inhibits the HR pathway by direct binding to RAD51, therefore affecting its assembly with ssDNA, which is observed in reduced RAD51 foci formation [8]. Also, they demonstrated a synthetic lethal interaction of Polθ and the HR repair pathway in HR-deficient ovarian tumor cells, which revealed that depletion of both Polθ and HR leads to cellular death.

In addition, in the research of Goullet de Rugy et al. (2016) [49], siRNA was used to perform knockout of Polθ and genes encoding enzymes involved in DNA metabolism, i.e., *FANCA, RECQL5, MUTYH, NEIL1,* and *USP22*, to check synthetic lethal interaction between them, in model of colorectal cancer cells. Mentioned genes were selected in the screen, also performed with use of siRNA in the cells with Polθ overexpression. The study did not show significant changes in cells viability after double knockout, versus cells without Polθ depletion. Therefore, it is possible to assume that Polθ does not have synthetic lethal correlation with any of these genes. Although, the scientists treated the Polθ knockout cells with hydroxyurea and cytarabine, drugs suppressing DNA replication fork progression and they exhibited increased sensitivity to the drugs, observed in decreased cell viability in comparison to the control. These results suggest that Polθ is involved in replication fork interruption [49].

Finally, in the studies conducted by Pan et al. (2021) [9], the shRNA mediated Polθ knockout was performed using a lentiviral vector with the purpose of analyzing Polθ significance in liver cancer cells (HCC). Successfully obtained knockdown led to decreased proliferation, migration, and metastasis, as well as increased apoptosis of cancer cells. Such effects may suggest that Polθ is involved in these processes and its inhibition may disturb the development of cancer cells. These results were also confirmed in vivo [9].

Presented studies show that Polθ depletion by itself also influences tumors survival, however compilation with different cytotoxic drugs or another DNA repair pathway deficiency increases the sensitivity of cancer cells to these agents. Therefore, in authors’ opinion, it is a good indication for potential use of Polθ inhibitors in clinics to use it not as a single therapy, especially given that there is a risk that tumors will develop resistance to Polθ inhibitors, similarly to what was observed when administrating PARPi.

### 3.2. CRISPR/Cas9 Technology

#### 3.2.1. Description of the Technique

The most recent technique to achieve gene modulation is CRISPR/Cas9—Clustered regularly interspaced palindromic repeats/CRISPR associated protein 9. This technique was designed based on a naturally occurring CRISPR/Cas system in prokaryotes, serving as an immune system, and defending them from foreign DNA particles of a viral or plasmid origin [38,39,50,51]. Three components are crucial in the case of CRISPR/Cas9 procedures, i.e., guide RNA, Cas9 nuclease, and target DNA with protospacer adjacent motif (PAM) [38,39]. Guide RNA, also referred to as single guide RNA (sgRNA), is a molecule that combines functions of two RNAs working in natural processes of bacteria, CRISPR-derived RNA (crRNA) and trans-activating CRISPR RNA (tracrRNA). Cas9 protein, derived from *Streptococcus pyogenes*, is guided by RNA and can target complementary fragments of DNA only when there is PAM motif, a short sequence (2–5 nt) on one strand of DNA [38,50,52]. The cooperation of sgRNA and Cas9 enzyme leads to a double strand break in the target sequence, which could be repaired by non-homologous end joining (NHEJ) or homology-directed repair (HDR) mechanisms (Figure 2). NHEJ repair occurs when there is no homology between created ends, and it usually generates knockout of the gene. However, HDR works while homology between ends occurs, which gives a chance to introduce an extra sequence to an existing one and create a knock-in [32,38,50]. It is additionally worth noting that the CRISPR/Cas system described above is of type II (out of three discovered types) and it needs only one Cas9 nuclease [38,39]. 

CRISPR can have various modifications and be used not only with Cas9 endonuclease (SpCas9), but also with other enzymes, e.g., Cas13, SpCas9, Cpf1, Cas12. Depending on the structure, the characteristics and application of the system may vary, for example Cas13 targets RNA instead of DNA [38,39]. CRISPR/Cas technology has a broad range of applications within gene editing, e.g., DNA and RNA editing, genome screening, live-cell imaging, virus and bacteria pathogen detection, inhibition, and killing, and gene therapy. It is also well developed in cancer research, e.g., in discovering the role of mutations in carcinogenesis by removing them from the genome, or creating cancer models by targeting specific cancer suppressor genes which lead to tumor formation, or by removing the genes that in consequence cause cancer cell death [39]. The application in cancer therapy was proven by Lu et al. (2020) [53] in phase I clinical studies where T-cells with *PD-1 gene* silencing done by CRISPR/Cas9 were administrated to the patients bearing non-small-cell lung cancer. The treatment did not cause adverse effect, howeverit did not stop cancer progression [53]. There is evidence that CRISPR/Cas9 could be used to overcome drug resistance in cancer cells [51].

#### 3.2.2. Application in Studies 

This technique has a huge therapeutic potential. However, it also raises some ethical controversies due to its ability to change human genome. Apart from its strongest advantages, such as versatility, easiness to reach the target DNA, relatively high efficiency and possibility to target multiple sites at once, CRISPR has its drawbacks [39,54]. The main one is the off-target effect and induction of uncontrolled changes in the genome. Moreover, there are problems with delivery in vivo and editing efficacy [51,54]. 

In the studies conducted by Schimmel et al. (2017) [28], with the application of CRISPR/Cas9 scientists were able to analyze, among others, TMEJ activity in mouse embryonic stem cells. The CRISPR technology was used to obtain knockouts of *POLQ, Ku80, LIG4* genes and double knockouts, respectively. Moreover, the site-specific blunt DSBs were introduced in marker gene *HPRT.* This allowed to measure how the mentioned knockouts affect the frequency of mutations in this gene, which are the result of mutation-prone repair, such as TMEJ. The results presented a decreased frequency of mutations in *POLQ* knockout cells compared to wild type cells, while *Ku80* and *LIG4* knockouts did not give any significant change in the mutation frequency compared to control cells. Moreover, in double knockout of *Ku80* and *POLQ*, the mutation frequency was even lower than in *POLQ* depleted cells alone. Furthermore, the authors measured sensitivity of the knockout cells to ionizing radiation. Both TMEJ- and NHEJ-depleted cells exhibited increased sensitivity, compared to wild-type, even though the changes in the mutation frequency were not observed in NHEJ knockouts. This analysis performed in the research made it possible to conclude that TMEJ, next to NHEJ, contributes to error-prone repair of DSBs in mouse embryonic stem cells. Secondly, in the case of an absence of the NHEJ repair mechanism, TMEJ can replace it completely, however not in reverse. Finally, TMEJ repairs DSBs with blunt ends and almost always requires microhomology near DNA break ends, which was measured in the presence of simple deletions induced by Cas9-WT in exon 2 and 3 of *HPRT* gene [28]. 

In their studies, Ferreira da Silva et al. (2019) [33] examined the role of NHEJ and Polθ-mediated a-EJ by inducing DNA breaks and knockouts with CRISPR-Cas9. The research showed that NHEJ is the main repair pathway to repair Cas9-induced DSBs. This study also confirmed that Polθ-mediated repair can substitute NHEJ when it is not present in cells [33].

Further studies carried out by Mateos-Gomez et al. (2017) [7] showed the application of CRISPR/Cas9 as well as shRNA in gene editing in mouse embryonic stem cells. Due to the CRISPR/Cas9 knockout of the helicase and the polymerase domains of Polθ separately, the scientists could prove their role in DSB repair, highlighting that the helicase domain favors Polθ-mediated repair by removing RPA. One of the measured parameters was frequency of chromosomal translocation, which is assumed to be caused by Polθ activity. Lower frequency of translocation was reported when the helicase or polymerase domain was depleted, which could lead to a conclusion that both are important for DNA ends joining. Next, the authors observed increased accumulation of IR-induced RAD51 foci regardless of which domain was depleted in cells. Moreover, with use of the CRISPR/Cas9 they performed HR-mediated gene targeting assay, revealing that both domains interact in HR suppression. In addition, the usage of shRNA allowed to achieve additional *BRCA1* gene knockout in the studied cells. In comparison to wild type cells, both types of double-knockout cell lines, i.e., without either helicase or polymerase domain and *BRCA1*, exhibit decreased growth. This experiment shows that helicase and polymerase activity of Polθ is necessary for HR-deficient cells. In these assays, cells lacking a central domain which interacts with RAD51 did not differ from wild type cells phenotype [7]. 

Moreover, Zhou et al. (2021) [13] used the CRISPR-Cas9 technology to knockout Polθ to compare it with novobiocin effects on human cells. The results of this study are further described below in Section 4.1.

Nevertheless, gene silencing is the most common application of CRISPR. The presented studies demonstrates that CRISPR-Cas9 is a powerful and versatile tool, which often brings better results than other methods of gene editing [38,55].

## 4. PolQ Inhibitors

The topic of Polθ inhibitors is still relatively new and not profoundly described in the literature. Within the last two years, few studies have indicated three potential candidates for Polθ inhibitors: novobiocin (NVB), ART588 with its isomers and RP-6685 [11,12,13]. 

### 4.1. Novobiocin

A coumarin antibiotic referred to as novobiocin, derived from *Streptomyces*, has been used to cure bacterial infections by attaching to the Bergerat fold present in the DNA Gyrase B’s ATP-binding site [56,57]. Novobiocin was introduced to cancer studies because of the similarity between DNA Gyrase and Heat shock protein 90 (Hsp90) structure [56]. Hsp90 is an evolutionarily conserved molecular chaperon responsible for maintaining over 300 client proteins, involved in crucial cell processes. Those proteins are also linked with ten hallmarks of cancer. Therefore, Hsp90 was placed as a target of anticancer therapy using NVB as its agent [57,58,59].

On the contrary to what was initially hypothesized, NVB was found to bind Hsp90 at C-terminal region and inhibit it allosterically, instead of Bergerat fold located at N-terminal ATP-binding site like in case of DNA Gyrase Moreover, it became the first C-terminal Hsp90 inhibitor that did not cause the heat shock response [56,60]. However, research disqualified NVB from antitumor activity due to its high half maximal inhibitory concentration (IC50) value of approximately 700 μM. Nevertheless, based on those results, several derivative compounds that could block the Hsp90 protein were discovered and synthesized [57,58,60,61]. Furthermore, the researchers tried to involve NVB and its derivatives in many other applications, e.g., neurological studies, as a treatment for neurodegenerative disease. Together, these discoveries could give some perspective for the studies on novobiocin as Polθ inhibitor, especially that Polθ share a similar structure to Hsp90 protein considering the helicase domain with ATPase activity. 

The studies on NVB targeting Polθ are performed independent on previous once and so far, three original papers about NVB as Polθ inhibitor have been published. The following section will summarize the most important findings of those studies [13,31,62]. To our knowledge Zhou et al. (2021) [13] were the first to introduce NVB to Polθ inhibition. They performed a broad-spectrum analysis (small-molecule screening, secondary screening in the presence of ssDNA, P-based radiometric ATPase assay, dose–response and binding capacity experiment, thermal shift assays, molecular docking), which revealed that novobiocin as a specific inhibitor that binds directly to the helicase domain with ATPase activity, in vitro. Moreover, referring to the previous application of NVB, the scientists excluded its off-target activity on HSP90 and TOP2, a eukaryotic homolog of DNA Gyrase, suspected of being responsible for the cytotoxic effect of NVB in HR-deficient cells. Research proves that NVB particularly targets Polθ in human cells, which was examined by creating Polθ-knockout cells with the CRISPR-Cas9 technology. These cells were more resistant to NVB treatment than wild type cells [13].

NVB binds to the helicase domain of purified Polθ protein. This domain is crucial when deciding whether DSBs will be repaired by HR or TMEJ. By its ability to dissociate RPA from resected DNA ends, it promotes the annealing of microhomologies, and in consequence the TMEJ pathway. Therefore, it is possible to assume that inhibition of polymerase domain with NVB allows RPA action and leads to increase end resection mediated by BLM/EXO1, which stimulate HR repair and block NHEJ at the same time [7,13]. In cells with nonfunctional HR, excessive end resection may occur, accompanied by RPA accumulation, which can lead to cell death. Additionally, the RAD51 accumulation is predicted to be correlated with redundant DSB end resection, however not in PARPi sensitive cells. 

The described mechanism is well visible in studies on tumor xenografts of Zhou et al. (2021) [13]. However, intensified end resection is also visible in PARPi resistant cells with HR-restoration or HR-proficient U2OS, after treatment with NVB. The possible explanation of this mechanism is that with continuous inhibition by NVB, HR may not be efficient enough, so over-resected DNA ends and nonfunctional RAD51 accumulate and become toxic for cells. Therefore, it is assumed that NVB could kill cells stimulating DSB end resection or ssDNA and RAD51 accumulation [13]. 

Zhou et al. (2021) [13] investigated if NVB has a similar effect on cells as Polθ silencing with other methods, such as siRNA knockdown. However, they demonstrated weaker RAD51 and H2AX foci formation after ionizing radiation (IR) in NVB treated cells than it was confirmed in the studies of Ceccaldi et al. (2015) [8], where Polθ was knocked down by siRNA. These results indicate that NVB inhibits Polθ, leading to DNA repair impairment. Nevertheless, the effect might be weaker than inhibition achieved by siRNA.

In an animal model of mice with transplanted genetically engineered BRCA1-deficient (BRCA1^−/−^) breast cancer, the animals treated with NVB had significantly smaller tumors and lived almost three times longer than those treated with vehicle. In the next models of mouse xenografts with FANCF-deficient and proficient ovarian cancer cell lines, the group observed NVB effectiveness especially on FANCF-deficient tumors when the vehicle had an impact on any of the cases. RAD51 foci were also generated in NVB-treated tumors [13]. 

Further, in vitro tests show that NVB significantly decreased the survival of BRCA1^−/−^ and BRCA2-deficient (BRCA2^−/−^) RPE1 cells and generates the apoptosis in comparison to WT cells. Moreover, it induces DNA damage (chromosomal aberrations and radial chromosomes) at a similar level as cytotoxic drug mitomycin C. 

What should not be neglected in the context of Polθ inhibitors are PARP inhibitors and PARPi resistance, one of the reasons for the studies on Polθi. Therefore, Zhou et al. (2021) [13] examined the synergic activity of NVB, olaparib and rucaparib in HR-deficient cells. A stronger effect of PARPi together with NVB than alone in HR-deficient cells was demonstrated, and additionally NVB decreases the IC50 value of both PARP inhibitors in BRCA1^−/−^ and FANCF-deficient cells [13]. 

There are different hypotheses on the mechanism in which cells acquire PARPi resistance. Moreover, it is possible that Polθ is involved in this mechanism and its inhibition could resolve this problem [11,19,63]. Research shows that NVB can deal with not only one PARPi resistance mechanism. Zhou et al. (2021) [13] created clones of BRCA1^−/−^ RPE1 (Human Retinal Pigment Epithelial-1) cells resistant to PARPi in at least two different mechanisms, i.e., replication fork stabilization and HR restoration visible via RAD51 foci accumulation. Interestingly, the BRCA1 re-expression was not observed, which was unexpected since the protein interacts with PALB2 and BRCA2 at DNA damage site, indirectly facilitating RAD51 filament formation [64]. It was revealed that one of the clones exhibit lower expression of Shieldin complex component and the other clone decreased expression of 53BP1. Thus, a possible mechanism of HR repair resumption could emanate from the downregulation of the Shieldin complex and further NHEJ repair downregulation [65,66]. Importantly, all the clones kept comparable responsiveness to NVB as parental BRCA1^−/−^ RPE1, not resistant to PARPi. To prove that the NVB effect on cells comes from Polθ inhibition, the researchers genetically depleted Polθ in those clones and parental cells, as well as BRCA1 wild type cells. This influenced HR-deficient cells, which was visualized in a decreased survival rate but not wild type RPE1 cells, leading to conclusion that Polθ inhibition is the most effective in HR-deficient cells. Similarly, two cancer cell lines derived from patients with PARPi-resistance obtained via two different mechanisms, described above, were sensitive to NVB treatment, while the resistance to olaparib lasted. Moreover, after insertion of wild type BRCA1 cDNA to cells, *POLQ* expression and NVB sensitivity were lower. The results described above may lead to a conclusion that HR-deficient cells do not develop cross-resistance to NVB and PARP inhibitors. Moreover, said PARPi resistance mechanisms are independent on BRCA1 and most probably depend on Shieldin complex functioning [13]. 

However, Zhou et al. (2021) [13] discovered that NVB cannot omit each mechanism of PARPi resistance, namely, *BRCA2* gene somatic reversion. The BRCA2-deficient cells, with acquired PARPi resistance via this mechanism, did not react either to PARPi or NVB. The mentioned results were reflected also in vivo in patient-derived xenografts. Therefore, there is no clear evidence that Polθ plays a role in PARPi resistance, at least a mechanism is not yet known. 

As mentioned above, authors of various studies claim that *POLQ* mRNA expression is upregulated in HR-deficient cancer cells [8,9,10,26,27]. Research proved that elevated expression of POLQ mRNA and protein is specific for HR-deficient cells such as BRCA1^−/−^ cells, and it is correlated with cell sensitivity to NVB, since BRCA1^−/−^ PARPi resistance cell lines also exhibit higher *POLQ* mRNA expression. These observations were also confirmed in vivo in HR-deficient patient-derived xenograft models. Moreover, the cells, which develop PARPi resistance by *BRCA2* somatic reversion, expressed low levels of Polθ, which could indicate that this protein is not necessary for them. In conclusion, Polθ expression could serve as a biomarker of responsiveness to NVB and could be applied in the treatment for patients in the future [13]. 

To conclude, the publication of Zhou et al. (2021) [13] is the first to present studies on NVB in the role of Polθi, in vitro and in vivo, establishing NVB IC50 value at the level of 100 μM. It highlights the promising role of NVB in killing HR-deficient cells compared to wild type cells. Moreover, NVB enhances the cytotoxic effect of PARP inhibitors in the said cells. Most importantly, this study assumes that NVB can be used either alone or in combination with PARPi to deal with HR-deficient tumors, even in the case of developed PARPi resistance. The research shows that NVB preferentially kills HR-deficient cells both in vitro and in vivo [13]. 

Similar results were observed in studies of Patterson-Fortin et al. (2022) [62] who used DNA-PK inhibitor, namely peposertib. Performed CRISPR screening revealed that depletion of *POLQ* sensitizes cells to this inhibitor. Moreover, cancer cells with DNA-PK depletion achived in two ways by knockout or treatment with peposertib show upregulated level of Polθ and consequently revealed hypersensitivity to NVB, showing synthetically lethal interaction between these two repair mechanisms. The inhibition of Polθ by NVB and DNA-PK with peposertib induces a toxic level of DSB end-resection. This effect was shown and confirmed in increased RPA, BrdU, γH2AX foci, and ssDNA fragments. Further analysis, which supports the results provided above, showed enhanced RAD51 foci accumulation, increased DNA damage visualized in comet assay and induction of apoptosis. Therefore, this research presents evidence that the inhibition of both TMEJ and NHEJ repair pathways leads to excessive end resection, and in consequence cell death [62].

Another experiment also proved the convergent effect of NVB, peposertib and *TP53* knockout. The cells with *TP53* knockout revealed increased sensitivity to NVB and, in combination with peposertib, it significantly lowers its possible used dose. This mechanism was correlated with increased Polθ expression. Presented results were confirmed also in patient-derived ovarian cancer organoids with *TP53* mutations, showing a drop in viability due to the toxic level of DSB end-resection. What is more, the combination of the treatment with NVB and peposertib leads in vivo to a decrease of tumor growth in mice. Although NVB influence tumor growth alone, together, the inhibitors demonstrate a stronger effect [62]. 

In conclusion, all findings demonstrate synthetical lethality between Polθ and DNA-PK, the crucial protein of the NHEJ pathway, as well as potency of their dual inhibition in cells lacking *TP53.* Moreover, cells lacking *BRCA1* and *BRCA2* also exhibited hypersensitivity to the combination of NVB and peposertib, which may suggest that not only Polθ is important for HR-deficient cells survival, but also DNA-PK. This indicates a next step in the development of cancer treatment based on Polθ inhibition, particularly with novobiocin [62]. 

In other studies, researchers apply NVB to inhibit Polθ in cancer cells HCT116, as well as create Polθ knockout by CRISPR, method that was mentioned above in chapter 3 [31]. They used NVB to investigate additional Polθ activity in intra-chromosomal fusion generated by TALEN. The research proved discriminatory Polθ inhibition and reduction of EJ repair by half with an NVB dose of 100 μM. The same dose of NVB did not impact the viability of Polθ depleted cells. However, it significantly decreased the viability of WT cells. On the other hand, NVB did not reduce the frequency of intra-chromosomal fusion in WT cells, but it did only in the cells with changed *POLQ* expression, both surplus and deficient. Therefore, the authors assume that this process might be regulated by mechanisms dependent and independent on Polθ, leading to the conclusions that NVB may have an influence on cells by targeting not only Polθ [31].

The recruitment of the compounds targeting Polθ helicase domain, similarly to NVB, in oncologic patients with HR-deficiency is reported by Ideaya Bioscience (San Francisco, CA, USA), (https://www.ideayabio.com/pipeline/; accessed on 20 October 2022) [13,67]. The past experience of introducing NVB to clinical trial with rather poor results was not very encouraging. However, then, NVB application was not combined with any DNA repair deficiency. Nevertheless, it paves the way for future research [68]. 

### 4.2. ART558

Similarly to NVB, only three original papers using ART558 as Polθ inhibitor have been published so far and only several reviews mention it [4,11,18,21,24,32,62,67]. The first one to report were Zatreanu et al. (2021) [11] carried out screening of around 165,000 inhibitors against Polθ polymerase activity. Based on that assay, ART558, a small molecule inhibitor, was selected with most suitable results of the IC50 value (7.9 nM), solubility, and LogD [11]. 

It is worth pointing out that ART558 has a different mechanism of action compared to NVB since it targets the polymerase domain of Polθ. It binds to the allosteric binding site of the Polθ polymerase catalytic domain and enhances Polθ thermal stability in the presence of DNA. An isomer of ART558, namely ART615, was also discovered. However, this compound shows poor Polθ inhibition at higher concentration than in case of ART558 (at 12 µM). Therefore, it was used as a control compound to ART558 in the study of Zatreanu et al. (2021) [11]. It was also demonstrated that ART558 is specific to polymerase theta because it does not inhibit other polymerases, such as Polα, Polγ, Polη, and Polν, and any other kinases, including PARP1 and PARP2, even at 10 μM concentration [11].

Polθ inhibition by ART558 exhibits a synthetically lethal effect with HR repair genes, such as BRCA2, similar to the inhibition via siRNA. A model of BRCA2^−/−^, cells resistant to the PARP inhibitor, was used to visualize the mechanism. The cells reveal sensitivity to ART558, but not to ART615. Furthermore, similarly to genetic silencing of Polθ, ART558 treatment with olaparib disturbs BRCA2^−/−^ cells survival, confluency and induces apoptosis much stronger than in wild type BRCA2-proficient cells. In addition, the scientists observed that ART558 induces several events related to DNA damage and its level is higher in the knockout cells versus wild type, including accumulated lasting γH2AX foci elevated micronuclei formation and chromosomal abnormalities [11]. 

Responsiveness of BRCA2^−/−^ cells was also proven in a model of CAPAN1 (pancreatic ductal adenocarcinoma tumor) cells derived from the tumor with naturally occurring BRCA2 mutation. Based on the application of these cells and their modification with a restored open-reading frame of BRCA2, the research shows significantly lower sensitivity of the cells with restored BRCA2 to ART558 compared to the BRCA2^−/−^ cells. What is more, it was confirmed using genomic databases that CAPAN1 appeared to be one of the most sensitive cell lines to Polθ inhibition among other 249 BRCA-deficient tumors [11].

Along with BRCA2, a dual synthetically lethal effect was demonstrated between Polθ inhibition by ART558 and PARP by olaparib in BRCA1^−/−^ RPE1 cells. Meanwhile, ART558 in the same concentration, which influenced knockout cells, had a minimal effect on normal human mammary epithelial cell lines or *BRCA*-gene wild type triple-negative breast tumor cells. ART558 sensitivity was also confirmed ex vivo in tumor organoid derived from BRCA1-mutant breast cancer, which was also sensitive to olaparib. The sensitivity was observed as decreased surviving fraction compared to BRCA1 wild type organoids. The presented results highlight the synthetically lethal interaction between Polθ and BRCA1 or BRCA2 [11].

In order to recognize other factors that could sensitize cells to mentioned inhibitors, the researchers conducted chemosensitization screens to ART558 and olaparib with the use of siRNAs in BRCA1^−/−^ and wild type RPE1 cells. In case of ART558 in wild type cells, siRNAs targeting the following genes caused sensitivity: *BRCA1, PALB2, POT1* and *POLH*. *BRCA1* and *PALB2* also appeared in olaparib sensitivity screening, which could be expected due to their role in HR. However, the role of *POT1* and *POLH* genes is not clear in this mechanism. *POLH* encodes polymerase η which is involved in translesion synthesis, and its overexpression is correlated with shorter survival of patients with lung cancer, similarly to Polθ [69]. On the other hand, *POT1* encodes one of Shelterin proteins, responsible for telomeres protection and telomerase regulation, the crucial processes for cell survival [70]. Thus, increased sensitivity to ART558 in absence of POT1 may arise from the mechanism independent on DNA repair. In BRCA1^−/−^ cells, the most important observation was that siRNAs targeting genes encoding proteins from the Shieldin complex induced sensitivity to ART558, which was not reported in cells with BRCA1. Also, genetic screens in mice with *POLQ* knockout revealed such a correlation between components of the Shieldin complex and Polθ depletion. The cited results may suggest that ART558 could be used to overcome PARPi resistance acquired by depletion of Shieldin complex elements in BRCA1^−/−^ cells by a mechanism of dual synthetic lethality, which also agrees with the results obtained by Zhou et al. (2021) [11]. 

Establishing various cell models of gene knockouts and their compilation, i.a., *BRCA1, 53BP1*, and Shieldin components: *SHLD1/2/3*, with *POLQ*, it was found that it gives a synthetic lethal effect. Moreover, the cell models possessing above mutations separately, reveal sensitivity to ART558, whereas staying resistant to olaparib [11].

The promising results of in vitro studies were shadowed by the fact that ART558 exhibits low in vivo metabolic stability in rats microsomes. Thus, if ART558 is to be introduced in clinical trials, this issue must be resolved. Nevertheless, the authors used another inhibitor, ART812, in the part of in vivo studies. It is important point for improvement for this inhibitor. Further, it was observed that tumors established in the rats with introduced double knockout BRCA1 and SHLD2 breast cancer cells were significantly smaller after treatment with ART812 [11]. 

Patterson-Fortin et al. (2022) [62], mentioned above in the context of novobiocin, also applied ART558. The results were consistent with the one obtained for NVB and confirmed the synthetically lethal relationship of Polθ and DNA-PK, while demonstrating increased cytotoxicity during the treatment with both inhibitors, ART558 and peposertib [62].

Other research group used ART558 in the treatment of transformed mouse embryonic fibroblast cells in extrachromosomal assay [24]. The intention of the experiment was to evaluate role of PARP1 and its inhibition in TMEJ. The study confirmed that a fully depleted function of PARP1 has a merely moderate effect in TMEJ disruption, which may help to understand why double inhibition of Polθ and PARP has a greater impact in the treatment of HR-deficient cancer cells [24]. 

The cited studies summarize the application of ART558 as a Polθ inhibitor. ART558 has a potential to be applied in cells resistant to PARP inhibitors and could be used in therapy alone or in combination with PARPi. Moreover, research proved the synthetically lethal interaction between Polθ and HR repair mechanism [11,24]. In addition, the derivative of ART812, ART4215, was introduced to clinical trials by the pharmaceutical company Artios Pharma Ltd. (Cambridge, UK). However, there is still much work that needs to be done to develop a sufficient therapeutic method based on the use of this inhibitor [11,18,32,71].

### 4.3. RP-6685

Recently, research on a new Polθi, RP-6685, was published by Bubenik et al. (2022) [12]. The authors discovered, synthesized, and characterized, via multiple biopsychical methods, a potent, selective, and orally bioavailable inhibitor of Polθ polymerase domain, similarly to ART558. The compound in in vitro and in vivo tests on cancer cells and mouse xenograft models, also HR-deficient, gives promising results. HEK293 LIG4-deficient cells exhibit decreased activity of Polθ-mediated repair pathway, after the treatment with RP-6685. Moreover, BRCA2^−/−^ HCT116 cells revealed a lower proliferation rate caused by RP-6685 treatment. In addition, the mice model with the BRCA2^−/−^ xenograft showed decrease of the tumor growth after first eight days of inhibitor administration, compared to vehicle. However, this effect did not last till the end of the 21st day of treatment. Nevertheless, the publication is rather focused on physicochemical characterization. Therefore, in our opinion, broader research is necessary for this inhibitor [12].

## 5. Conclusions and Prospects

Polθ may play a significant role in the human organism and be even more important in tumors. The level of its expression is elevated in cancer cells, while the depletion of Polθ achieved in various ways, genetically or chemically, leads to cancer cell death, especially in HR-deficient cells [8,9,10,11,13,62]. It is therefore difficult to distinguish the most efficient strategy to inhibit or knockout Polθ protein. However, with increasing knowledge and development, the CRISPR/Cas9 technology seems to be the most promising genetic method. Nevertheless, due to ethical issues, it is still not available for patient therapies, on the contrary to small-molecule inhibitors [38,39,55]. 

The discovery of Polθ and its inhibitors is undoubtedly a next chapter in cancer treatment. Nevertheless, Polθi research is in early stages and clinical studies are needed to prove their potency. There are high expectations that Polθi will be introduced into cancer therapies, however it is possible that cancer cells will also develop resistance to Polθi, similarly to the case with PARPi [11,13,24,31,51,62,72,73].

If Polθi eventually become registered anticancer drugs, combining them with inhibitors of other DNA-repair proteins such as PARP1, as well as using them in monotherapy could be tested in patients [11,13].

Attention should also be paid to the identification of biomarkers that could indicate tumors which are sensitive to Polθ inhibition. The level of Polθ expression itself could serve as a biomarker [13,62,73].

To summarize, Polθ inhibitors, such as novobiocin, ART558, and ART812, respectively, lead to the death of cancer cells both in vitro and in vivo, mostly in the case of HR-deficient cells. They induce biomarkers of DNA damage, such as RAD51 and RPA foci, γH2AX foci, or micronuclei formation, which may give satisfying results at the stage of pre-clinical research. Both small molecules, or compounds synthesized based on them, are being introduced in clinical trials. Therefore, many scientists are waiting impatiently for the results of the next steps of studies on NVB and ART558 as well as new candidates for Polθi [11,13,18,32,62,67]. Moreover, a newly discovered inhibitor, RP-6685, shows promising results in vitro and in vivo in reducing cancer development [12].

Polθ inhibitors and new Polθ synthetically lethal interactions are fast developing research topics. Results of research showing a new synthetically lethal interaction between novobiocin and DNA-PK inhibitor peposertib were published in August 2022 [62]. This illustrates the potential of the research in this topic and how much is still to be discovered.

## Data Availability

Not applicable.
